# Synergistic Approach to Radicular Cyst Management in an Adolescent: Utilizing Platelet-Rich Fibrin, Demineralized Bone Matrix Xenograft, and Type I Collagen Resorbable Membrane for Optimal Healing

**DOI:** 10.7759/cureus.90127

**Published:** 2025-08-14

**Authors:** Rohit Mishra, Debapriya Pradhan, Saurabh Tiwari, Varsha Choubey, Nikita Saini

**Affiliations:** 1 Department of Periodontics, Hitkarini Dental College and Hospital, Jabalpur, IND; 2 Department of Pedodontics and Preventive Dentistry, Hitkarini Dental College and Hospital, Jabalpur, IND

**Keywords:** fractured teeth, osseo bone graft, platelet-rich fibrin (prf), pus discharge, radicular cyst

## Abstract

Dentoalveolar injuries can lead to complications like pulpal necrosis, apical periodontitis, and usually radicular cysts, which are common inflammatory odontogenic cysts often triggered by trauma or infection. Radicular cysts are typically asymptomatic but may cause slow, noticeable swelling. This case report details the surgical management of the same in the periapical region of a maxillary incisor in a 12-year-old female patient. The approach included root canal therapy followed by periapical surgery. The cystic lesion was surgically enucleated, and a combination of platelet-rich fibrin (PRF) and demineralized bone matrix (DMBM) xenograft was applied to enhance healing, alongside a type I collagen resorbable membrane. Follow-up evaluations showed the patient was asymptomatic, with significant periapical healing and almost complete bone regeneration observed on radiographs.

## Introduction

Cystic lesions are a common type of chronic pathology, often remaining asymptomatic and only discovered incidentally. Radicular cysts are the most frequent among these, comprising over half of all jaw cysts. They can develop at any age and affect both sexes equally [1]. These cysts arise primarily from the epithelial remnants of Malassez in the periodontal tissue, with pulp inflammation being a key contributing factor [2]. These remnants multiply in response to inflammation triggered by bacterial antigens from necrotic debris and dead pulp tissue, typically resulting from caries or trauma to the tooth [3]. Their high prevalence, accounting for more than two-thirds of jaw cysts, underscores their significance in clinical diagnosis [2]. 

Radicular cysts often go unnoticed until they are detected through routine radiographic examinations, as they tend to remain asymptomatic in many cases. However, in some instances, long-standing lesions may lead to acute flare-ups, characterized by swelling, pain, and pus discharge. These exacerbations are usually the result of secondary infections or the expansion of the cyst, which can place pressure on surrounding tissues. Timely detection and a comprehensive approach to its treatment are essential to prevent such complications, as untreated cysts may cause further damage, including bone loss and displacement of adjacent teeth along with their major nerves and apical neurovascular bundles [4, 5].

This case report showcases the effective management of a radicular cyst through the use of platelet-rich fibrin (PRF) in conjunction with demineralized bone matrix (DMBM) xenograft and a type I collagen resorbable membrane, employing guided tissue regeneration (GTR) techniques. The combined regenerative properties of these materials significantly accelerate the healing of bony defects, demonstrating an effective approach to promoting rapid and robust bone regeneration.

## Case presentation

A 12-year-old female patient presented with the chief complaint of a broken tooth in the upper front tooth region for four to five years. The patient had no medical history and was not taking any medication. The patient's history indicated trauma to tooth 21, which occurred four to five years ago. It was accompanied by recurrent swelling and pus discharge. On intraoral examination, an Ellis Class II fracture was seen in relation to tooth 21. Grade 1 mobility with tooth 22 was noted. No intraoral or extraoral swelling/inflammation was present. Pulp vitality test negative for teeth 21 and 22, and also painless on vertical percussion. The patient was advised to undergo cone-beam computed tomography (CBCT) imaging.

**Figure 1:**
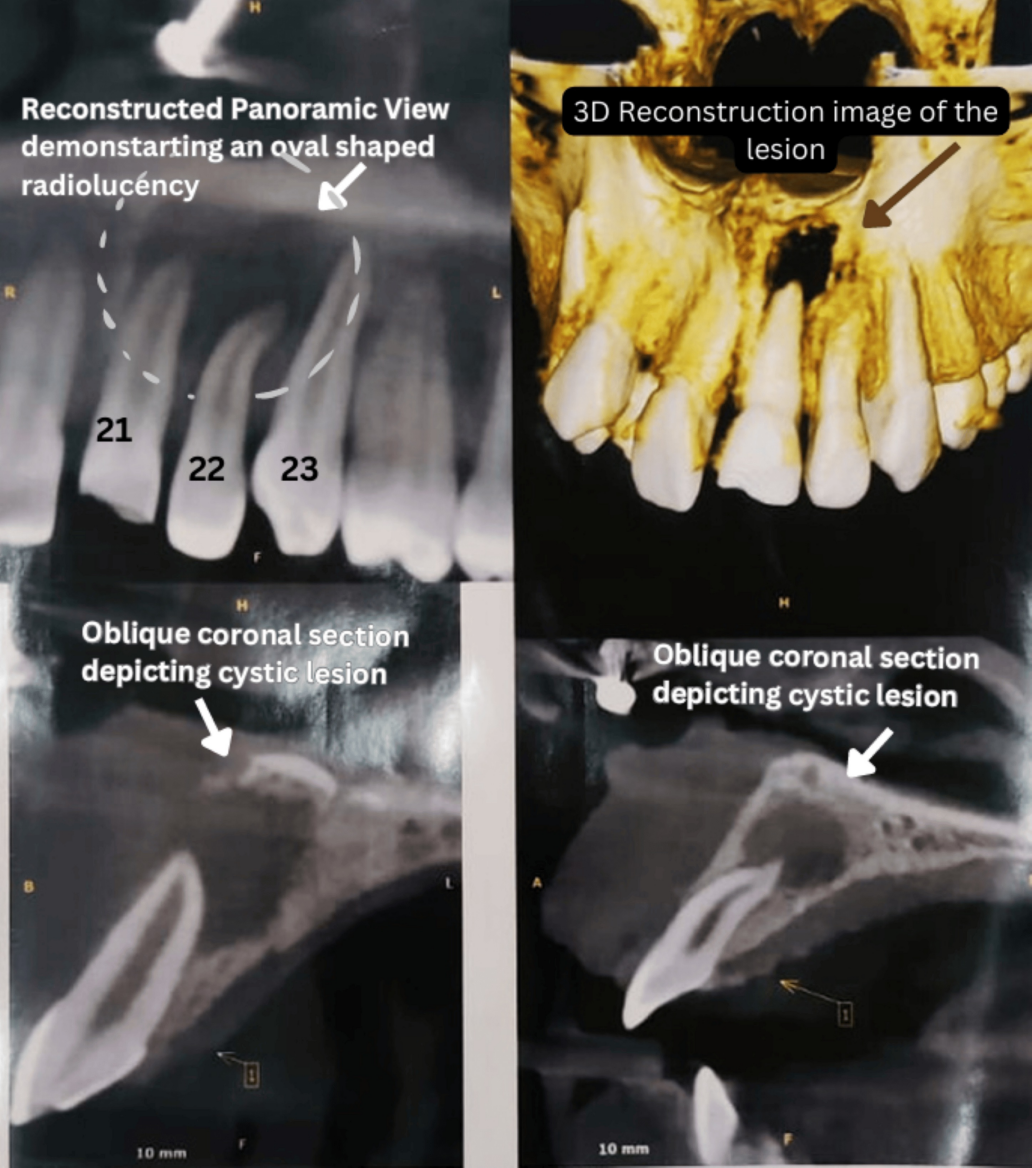
Cone-beam computed tomography imaging of the maxillary anterior region The reconstructed panoramic view of teeth 21, 22, and 23 reveals a cystic lesion at the root apices. A 3D CBCT reconstruction highlights its extent. The oblique coronal sections illustrate the lesion's relationship to surrounding structures and show thinning of the palatal bone and partial perforation of the labial bone.

It revealed a well-defined, oval-shaped cystic lesion with sclerotic borders involving the root apices of teeth 21 and 22. This explains the mobility seen in relation to tooth 22. The unilocular lesion extended mesiodistally from the midline to the palatal surface of tooth 23, though the apex of tooth 23 remained uninvolved. Mesially, the lesion bordered the cortical outline of the incisive canal, while superoinferiorly, it approached the nasal floor and palatal bone plate, both of which remained intact. It caused thinning of the palatal bone plate and partial perforation of the labial bone plate near teeth 21 and 22. The lesion's dimensions were approximately 11.6 mm labiopalatally, 11.2 mm mesiodistally, and 11.0 mm superoinferiorly (Figure 1).

Given the patient's history of traumatic injury to tooth 21, these findings, along with the lesion's characteristics, led to the diagnosis of a radicular cyst.

**Figure 2:**
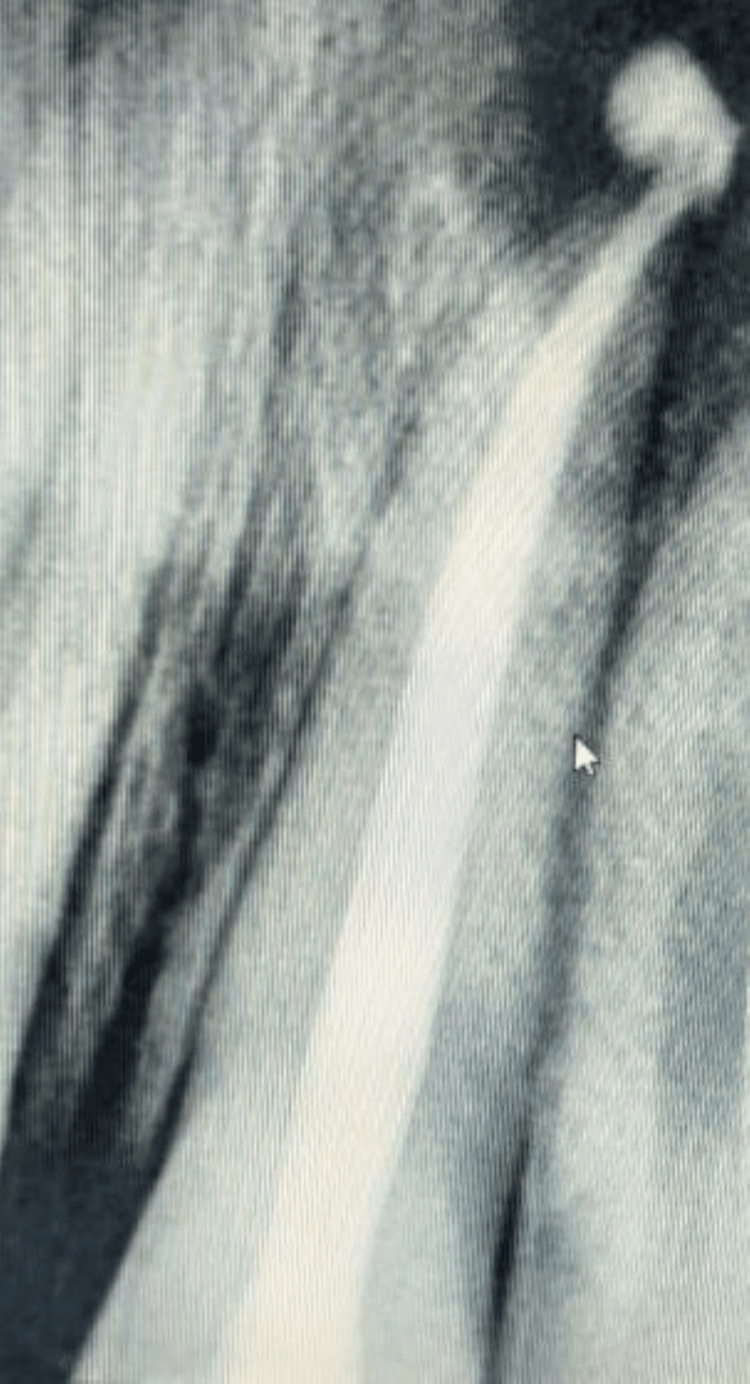
Post-obturation radiograph of tooth 21

A root canal treatment was initiated for tooth 21, followed by surgical enucleation of the lesion. Clinical and radiographic assessments suggested that the mobility of tooth 22, due to bone resorption, would improve as the lesion healed after treatment. The root canal procedure for tooth 21 was carried out under rubber dam isolation, with working length determination (19.5 mm) and completion of biomechanical preparation. Irrigation was performed using normal saline and 3% sodium hypochlorite, along with a diluted metronidazole infusion (500 mg/100 ml) in 0.9% sodium chloride to achieve complete disinfection. Additionally, calcium hydroxide was placed as an intracanal medicament. The patient was recalled after 14 days to complete the endodontic treatment. In a sterile environment, the canals were sealed with Prevest Denpro EndoSeal (Prevest Denpro Limited, Bari Brahmana, India), which offered additional benefits due to its anti-inflammatory, antiseptic, and germicidal properties. It was followed by successful obturation with gutta-percha (Figure 2).

**Figure 3:**
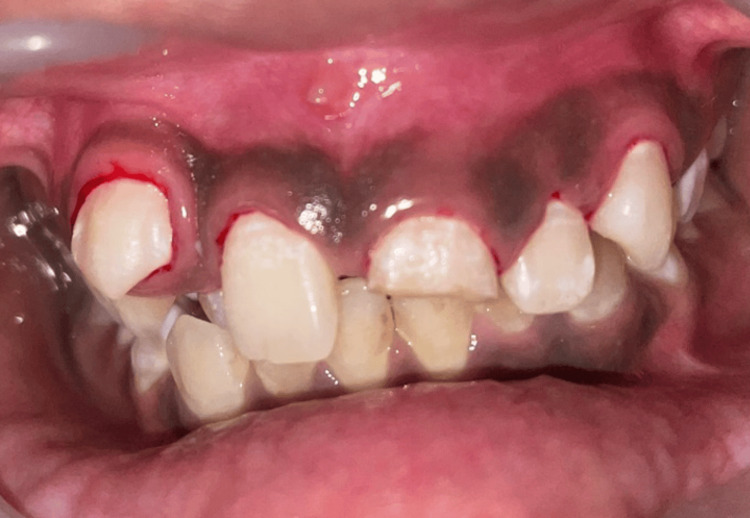
Frontal view of the teeth prior to the surgical procedure.

A composite restoration was then placed on tooth 21. Oral prophylaxis was initially performed with an ultrasonic scaler to ensure a clean area. Preoperative disinfection of both the extraoral and intraoral surfaces was carried out using a povidone-iodine solution (Figure 3).

**Figure 4:**
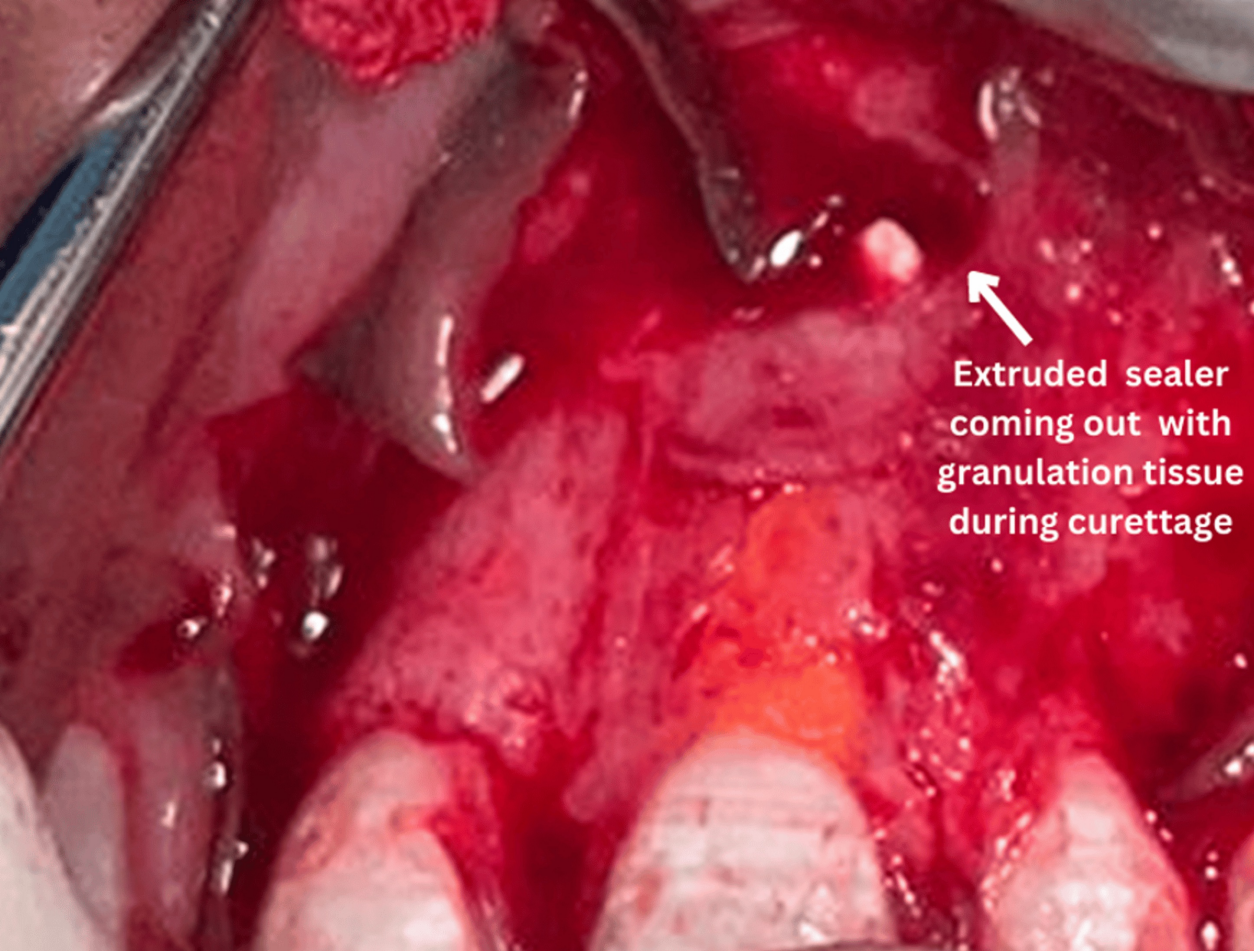
Curettage of the cystic lesion

Local anesthesia was administered, followed by crevicular incisions around teeth 11, 21, 22, and 23 using a surgical scalpel blade number 15. Releasing incisions were made distal to teeth 11 and 23. A full-thickness mucoperiosteal flap extending from 11 to 23 was then reflected with a periosteal elevator. The cyst was carefully curetted, and granulation tissue was excised. The extruded endodontic sealer also came out along with the diseased soft tissue (Figure 4).

**Figure 5:**
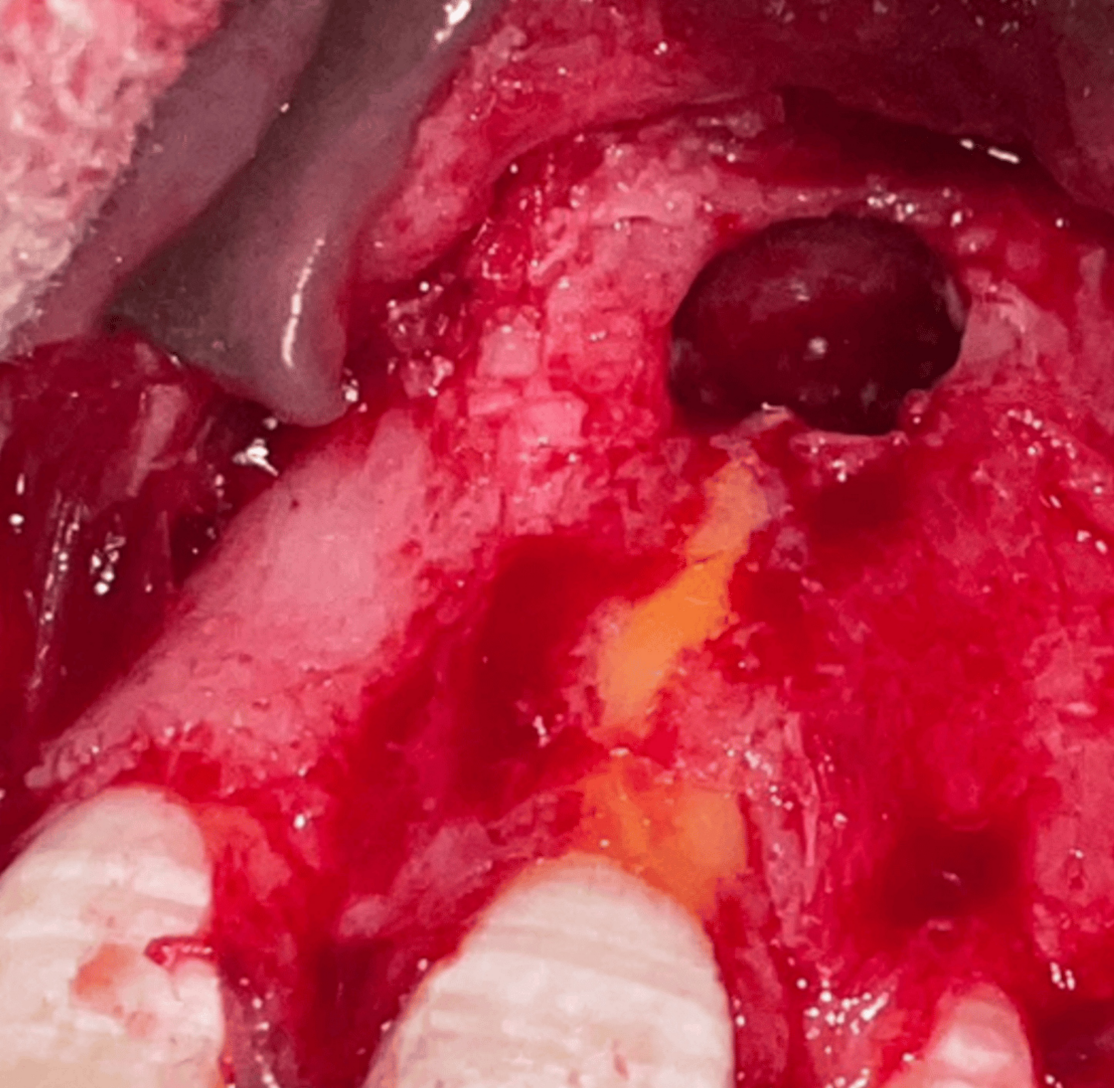
Photograph taken after irrigation and curettage of the cystic lesion

This was followed by a thorough irrigation of the bony lesion with Betadine and normal saline. The cavity was dried and examined for any remaining internal debris (Figure 5).

**Figure 6:**
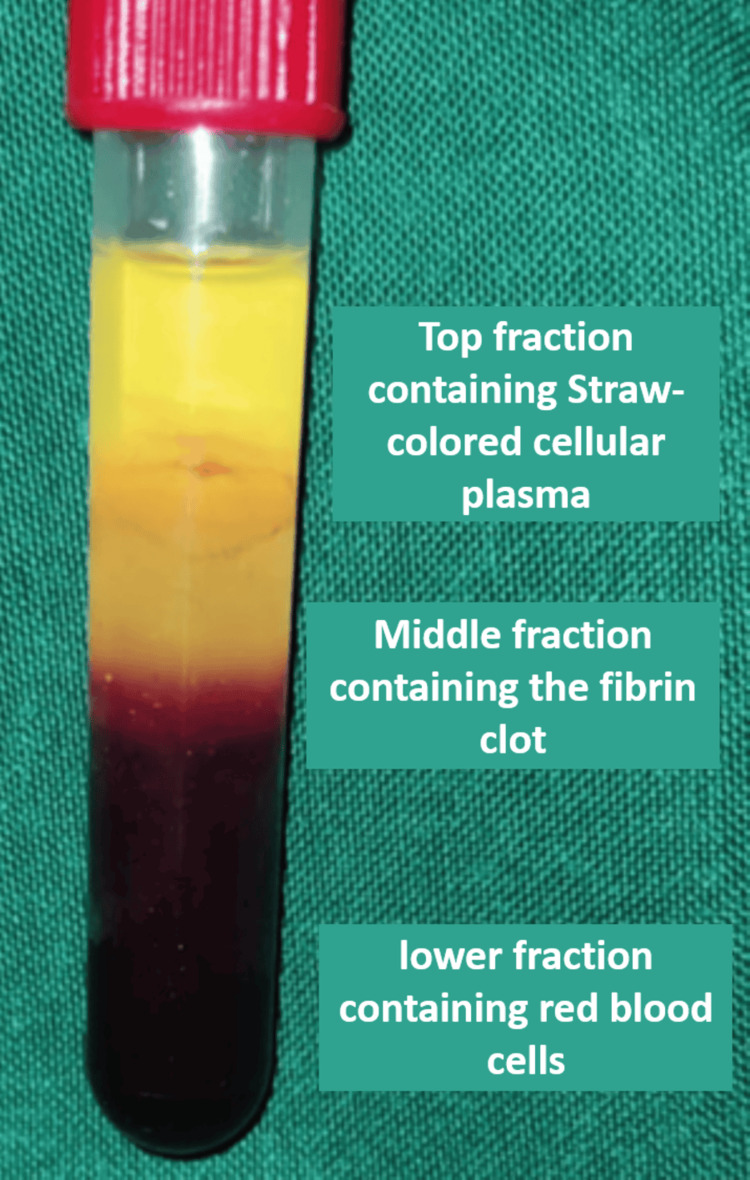
Prepared platelet-rich fibrin (PRF)

Whole venous blood was collected into a sterile Vacutainer tube without an anticoagulant. The tube was then centrifuged at 3,000 revolutions per minute (rpm) for 10 minutes. The straw-colored plasma was removed, and the middle layer, known as PRF, was collected 2 mm below the lower dividing line (Figure 6).

**Figure 7:**
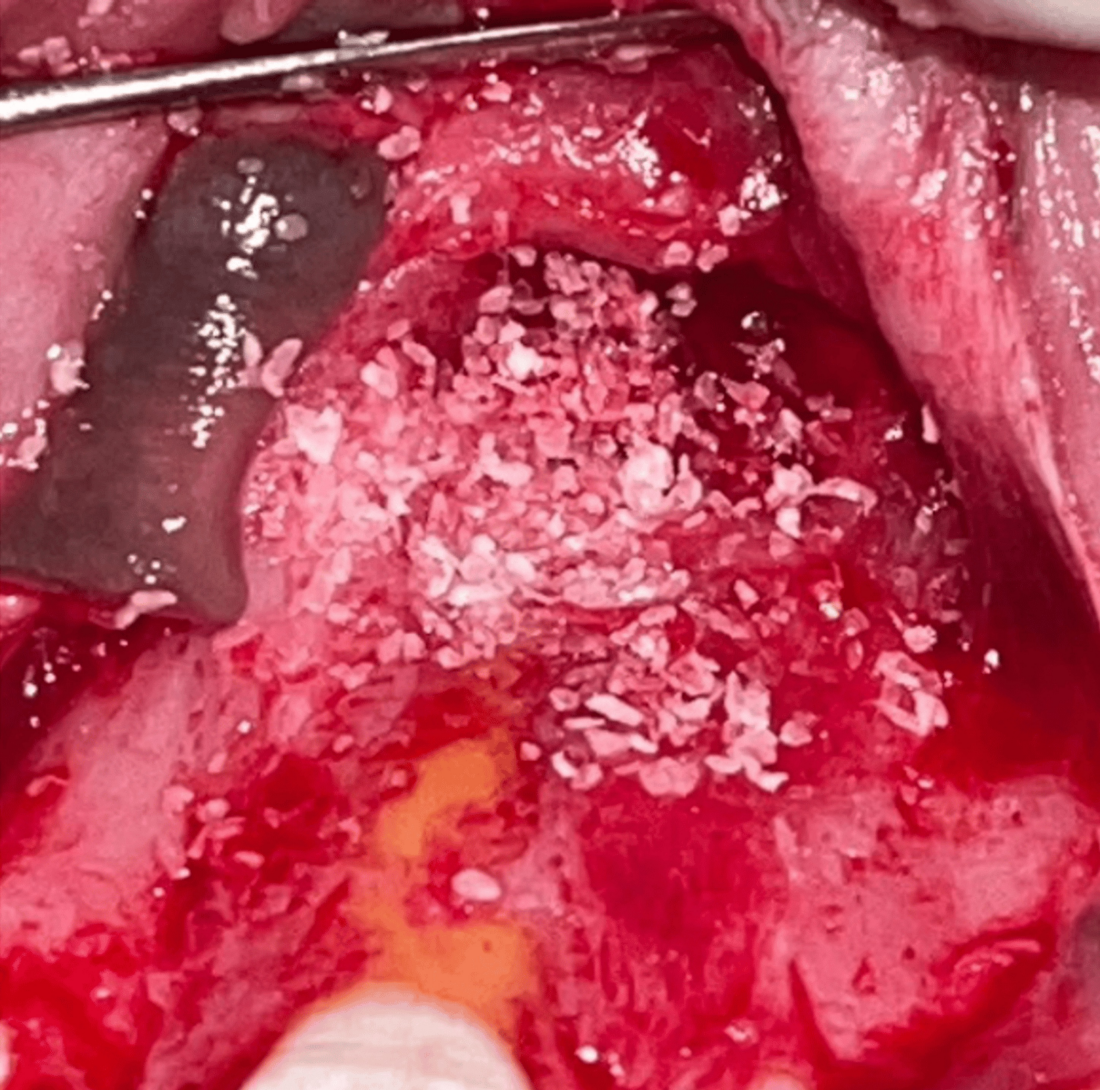
Bony cavity filled using platelet-rich plasma mixed with demineralized bone matrix xenograft (Osseograft).

This PRF was combined with DMBM xenograft, commercially known as Osseograft (Advanced Biotech Products Pvt. Ltd., Chennai, India), and used to fill the cavity completely (Figure 7).

**Figure 8:**
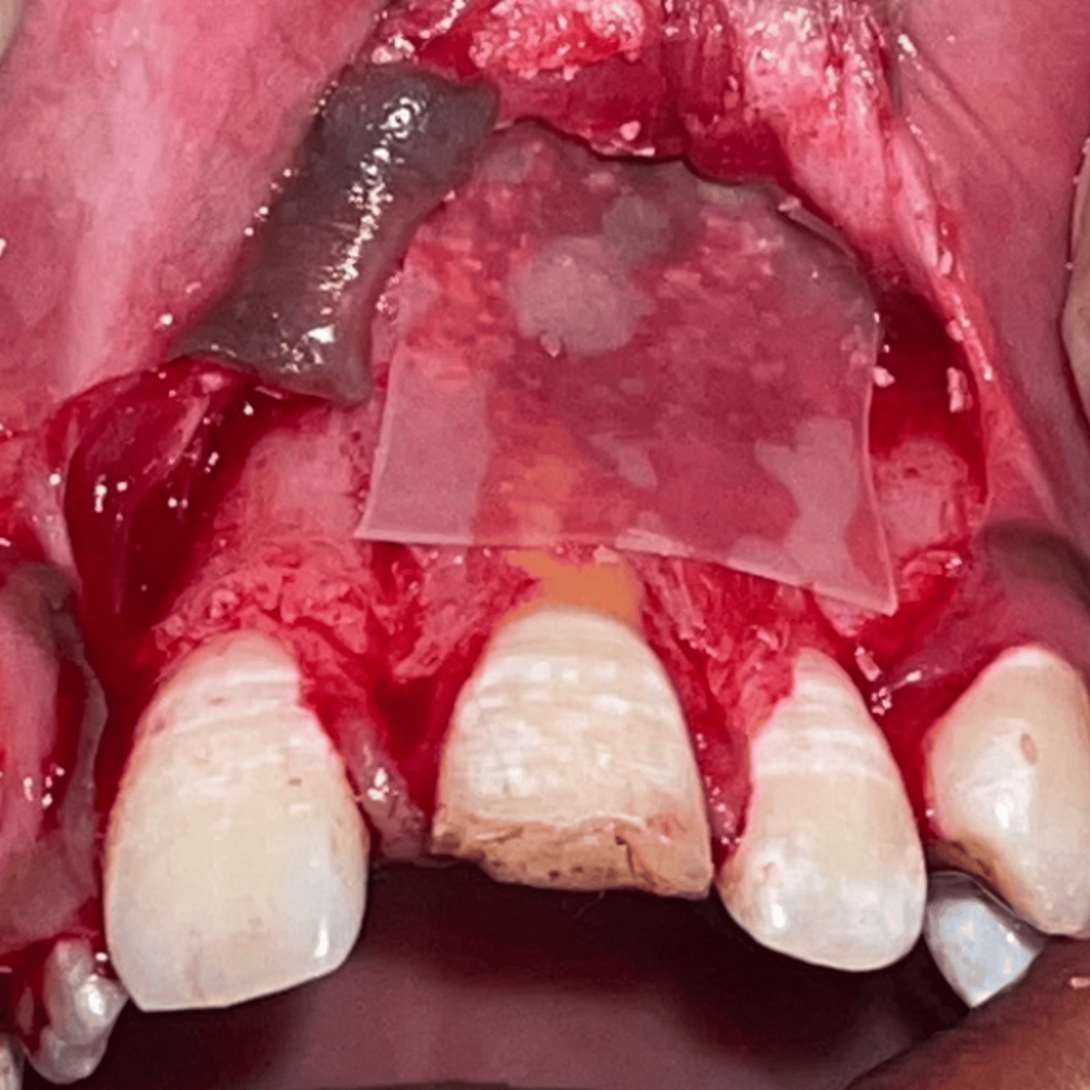
Healiguide, a guided tissue regeneration (GTR) membrane, placed in position.

A type I collagen resorbable membrane, commercially known as Healiguide (Advanced Biotech Products Pvt. Ltd.), was then positioned over the bone window (Figure 8).

**Figure 9:**
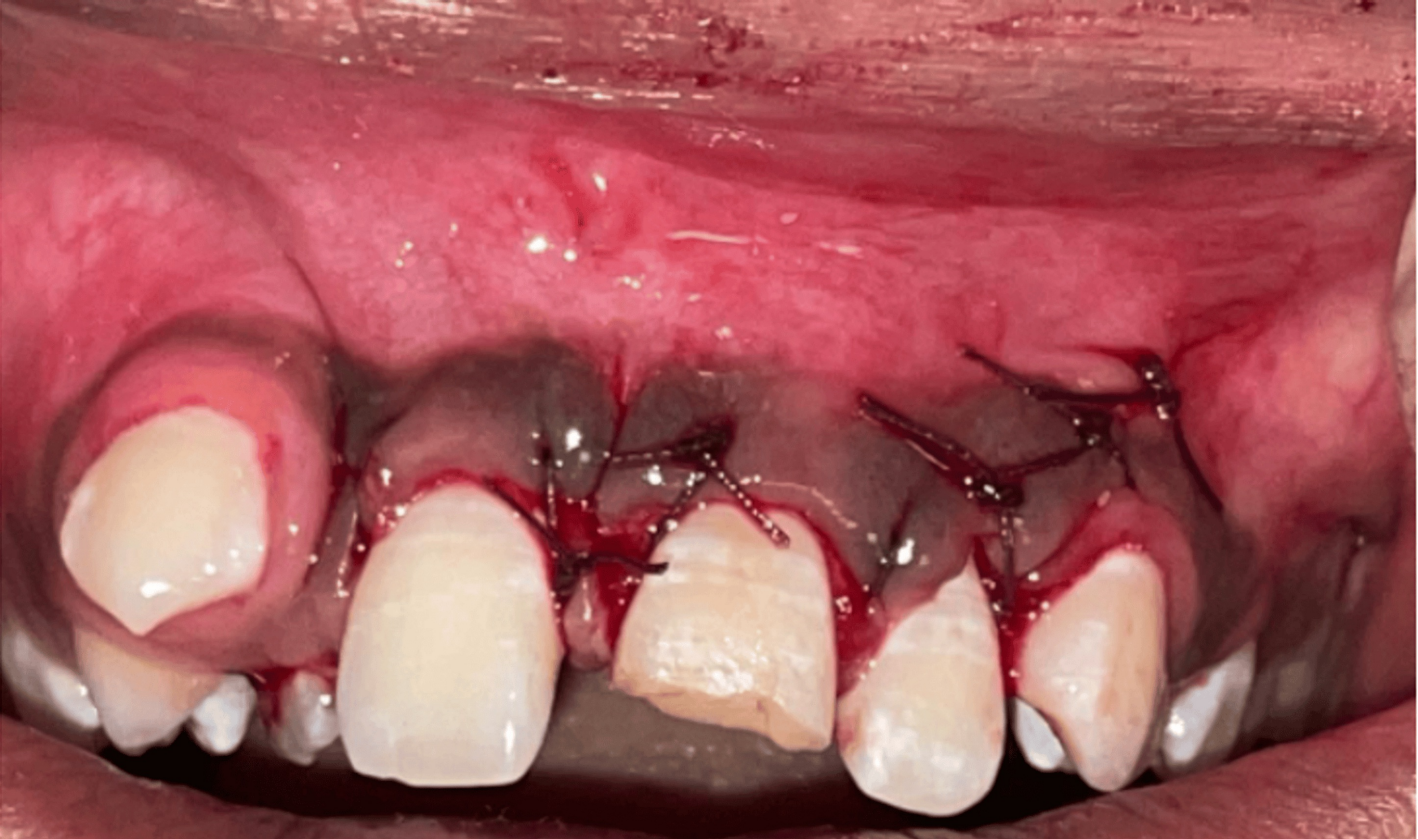
Sutures placed

The flap was adjusted to achieve a first-intention closure with no tension on the tissues. The suture selected was a 3-0 Vicryl absorbable braided suture, chosen for its ease of handling and excellent biocompatibility. Hemostasis was achieved effectively (Figure 9).

After the procedure, the patient was given instructions and prescribed antibiotics and analgesics for a week. The area was treated with 0.2% chlorhexidine gel for two weeks, and ice was applied for the first 72 hours. The biopsy collected during the surgery was analyzed, confirming the presence of a radicular cyst.

**Figure 10:**
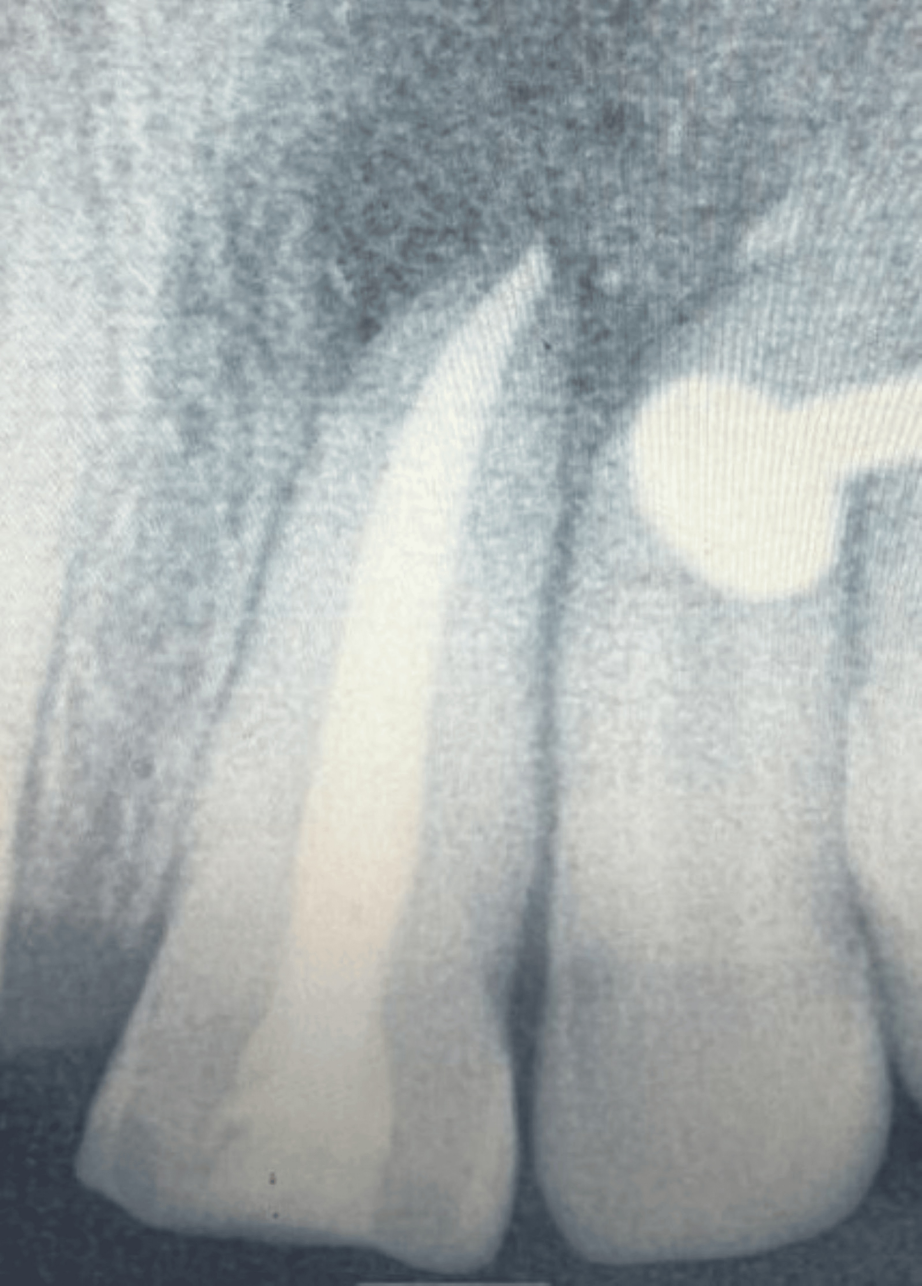
Radiograph taken on during the follow-up session at three weeks; a nosepin visible as an artifact.

The patient was scheduled for postoperative evaluation at three weeks. Radiographic examination revealed significant and satisfactory bone healing (Figure 10).

**Figure 11:**
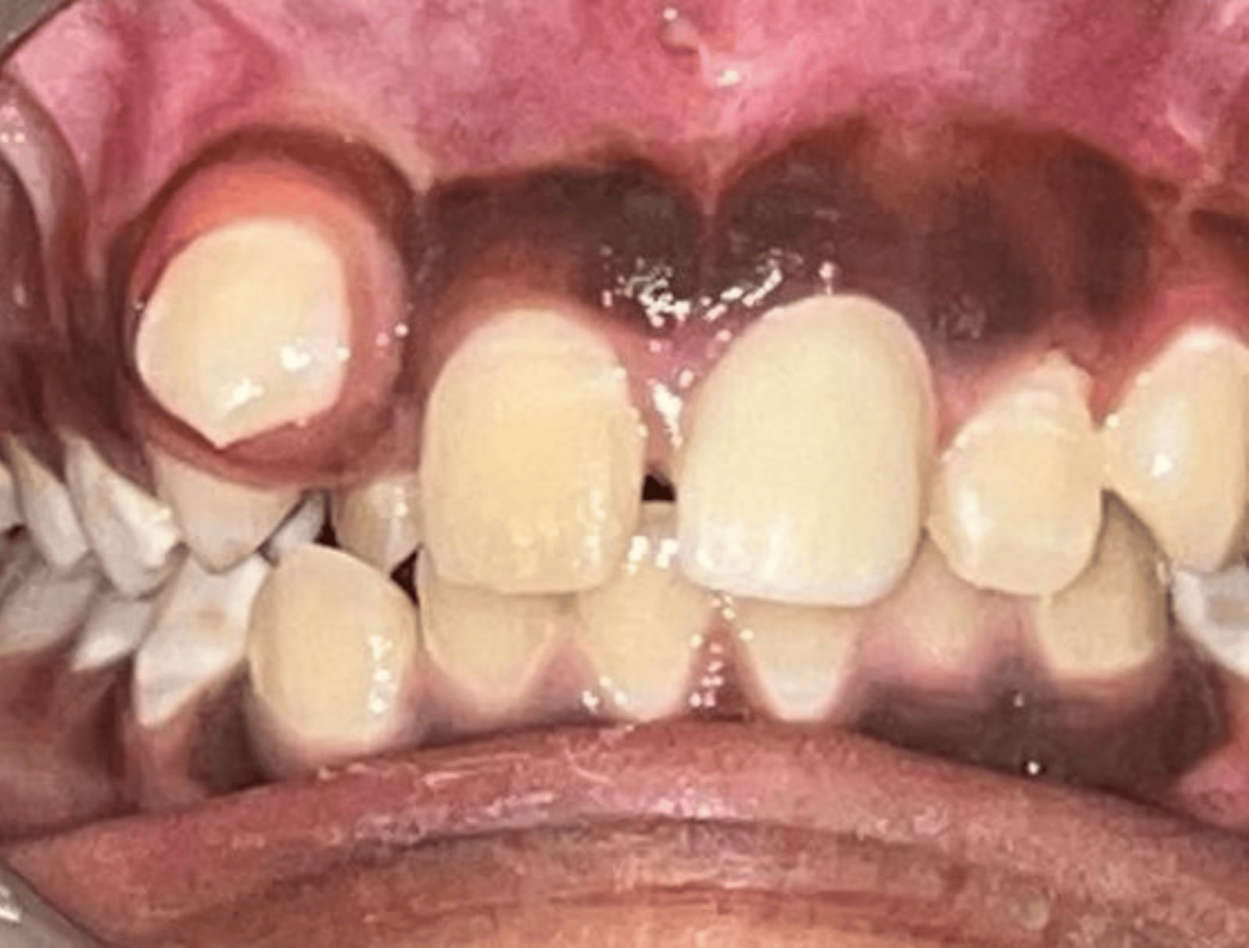
Tooth 21 after crown cementation

Also, when clinically examined, there was an absence of any mobility with tooth 22. Aesthetic rehabilitation of tooth 21 was completed by preparing the tooth for a zirconia crown, followed by its cementation (Figure 11).

**Figure 12:**
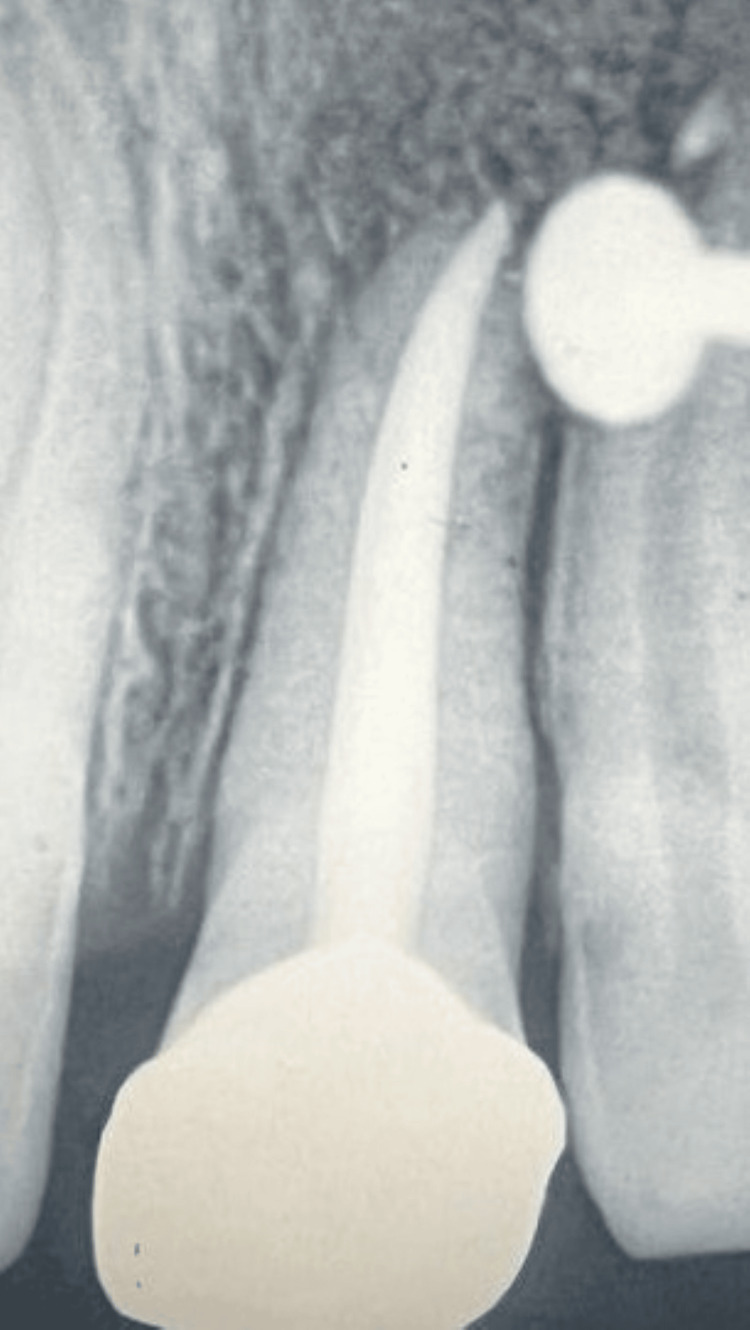
Radiograph taken during the follow-up session at 20 weeks; a nosepin visible as an artifact.

The patient was followed up at 12 and 20 weeks postoperatively. Radiographs taken at 20 weeks confirmed complete bone healing (Figure 12).

## Discussion

Radicular cyst management in adolescents demands a precise and integrative approach to ensure optimal healing and long-term success. This article explores a technique that integrates PRF, DMBM xenograft, and a type I collagen resorbable membrane, focusing on their synergistic effects in enhancing bone regeneration and repair.

While small radicular lesions often heal with endodontic therapy alone, larger lesions may require additional treatment [6]. In root canal treatment for teeth with chronic periapical lesions, utilizing root canal dressings between sessions is crucial for reducing bacteria that may be inaccessible to instruments or irrigation solutions, such as those residing in dentinal tubules and ramifications [7].

Given that the tooth in our case experienced trauma approximately five years ago, an intracanal medicament (calcium hydroxide) was used. Calcium hydroxide needs at least two weeks to demonstrate effective bactericidal activity, as determined by pH and calcium ion concentration analyses in the periapical area [7]. Consequently, the patient was scheduled for final obturation after 14 days of medicament placement.

But for extensive or multiple radicular cystic lesions, surgical options such as enucleation, marsupialization, or decompression, combined with endodontic treatment, are typically preferred [6]. Enucleation entails the total removal of the cyst and its surrounding sac, which reduces the risk of recurrence [1]. After thorough curettage and irrigation, techniques were employed by us to promote bone healing, specifically utilizing PRF and GTR methods. PRF, first introduced by Choukroun et al. in France, is a new generation platelet concentrate that simplifies processing and avoids complex biochemical blood handling, making it superior to platelet-rich plasma (PRP). PRF promotes wound healing, bone growth, graft stabilization, wound sealing, and hemostasis due to its well-organized fibrin matrix, which enhances stem cell migration and healing [8].

PRF is an ideal choice for interpositional applications, combining the ability to promote tissue repair with the function of a barrier that prevents the migration of unwanted cells. It inhibits early cellular invasion, releases growth factors, and forms a fibrin dressing that accelerates wound closure while supporting rapid healing of mucous membranes. Although its release of growth factors is slower than that of PRP, PRF demonstrates superior healing properties and shows strong potential as a supportive matrix for bone morphogenetic protein [8,9].

Complete recovery around the tooth apex requires regenerating the alveolar bone, periodontal ligament cells, and cementum. However, surrounding connective tissues may encroach upon the defect, hindering bone healing. GTR involves using a bone substitute material and a membrane that acts as a barrier to block non-bone-forming cells, such as epithelial cells, from entering the area. Additionally, GTR facilitates the migration of bone-forming cells to the defect, ensuring selective cell repopulation and directing tissue growth throughout the healing process [10]. To accomplish this, we employed DMBM xenograft (Osseograft) and a type I collagen resorbable membrane (Healiguide).

Osseograft, a DMBM xenograft made of type-I collagen, is used for regenerating osseous defects in periodontal and oral-maxillofacial surgeries. It acts as a bone substitute, providing a bioabsorbable matrix that aids in healing and stimulates new bone formation through its osteoconductive and osteoinductive properties. Additionally, it creates a scaffold that integrates bone-forming cells and blood vessels, fostering the development of healthy new bone and the repair of the defect [11].

Although PRF can serve a role similar to a GTR membrane, its primary limitation is its short resorption period of seven to 28 days, compared to the four to six weeks needed for most periodontal regeneration procedures. This rapid resorption can reduce its effectiveness in maintaining space [12]. These findings suggest that using both PRF and DMBM together in a bony cavity could significantly improve bone formation and are promising for effective osseous repair. The systematic review and meta-analysis by Theodosaki et al. also support that combining PRF with grafting materials accelerates bone healing in intrabony defects [13], a conclusion mirrored by our case.

Healiguide facilitates regeneration in bone defects by integrating into healing tissues or being broken down by macrophages within six to eight weeks; its collagen content promotes fibroblast migration and attachment through its scaffold-like structure, encourages platelet attachment, and enhances wound healing with its hemostatic properties [11]. Complete healing after periapical surgery usually requires about a year. However, the use of PRF speeds up this process, shortening the healing time to roughly six months [14]. Combining autologous PRF with bone grafts and collagen membranes presents promising potential for even more enhanced healing and functional recovery. In our case, the strategic application of these methods led to accelerated and improved healing, with significant progress noted within just five months.

## Conclusions

In conclusion, radicular cysts are frequently encountered in the oral cavity but often go unnoticed and rarely reach a size that is palpable. This case report demonstrates the effective management of a radicular cyst using a combination of endodontic treatment, enucleation, and well-selected biomaterials (PRF, DMBM xenograft, and a bioresorbable type-I collagen membrane). These approaches created a favorable environment for healing in both hard and soft tissues. However, as this is a single case report, the findings should be interpreted with caution, and further studies involving larger sample sizes are needed to validate the generalizability of this treatment strategy.
